# Generation and Characterization of a Zebrafish IL-2Rγc SCID Model

**DOI:** 10.3390/ijms23042385

**Published:** 2022-02-21

**Authors:** Robert Sertori, Realla Jones, Faiza Basheer, Leni Rivera, Samantha Dawson, Stella Loke, Somayyeh Heidary, Amardeep Dhillon, Clifford Liongue, Alister C. Ward

**Affiliations:** 1 School of Medicine, Deakin University, Geelong, VIC 3216, Australia; robert.sertori@hotmail.com (R.S.); realla.jones1@deakin.edu.au (R.J.); faiza.basheer@deakin.edu.au (F.B.); leni.rivera@deakin.edu.au (L.R.); samantha.dawson1@deakin.edu.au (S.D.); sheidary@deakin.edu.au (S.H.); amardeep.dhillon@deakin.edu.au (A.D.); c.liongue@deakin.edu.au (C.L.); 2 Institute for Mental and Physical Health and Clinical Translation, Deakin University, Geelong, VIC 3216, Australia; 3 School of Life and Environmental Science, Deakin University, Burwood, VIC 3125, Australia; stellaloke@gmail.com

**Keywords:** IL-2Rγc, immunodeficiency, zebrafish, microbiota, xenotransplantation

## Abstract

The IL-2 family of cytokines act via receptor complexes that share the interleukin-2 receptor gamma common (IL-2Rγc) chain to play key roles in lymphopoiesis. Inactivating IL-2Rγc mutations results in severe combined immunodeficiency (SCID) in humans and other species. This study sought to generate an equivalent zebrafish SCID model. The zebrafish *il2rga* gene was targeted for genome editing using TALENs and presumed loss-of-function alleles analyzed with respect to immune cell development and impacts on intestinal microbiota and tumor immunity. Knockout of zebrafish Il-2rγc.a resulted in a SCID phenotype, including a significant reduction in T cells, with NK cells also impacted. This resulted in dysregulated intestinal microbiota and defective immunity to tumor xenotransplants. Collectively, this establishes a useful zebrafish SCID model.

## 1. Introduction

The interleukin 2 receptor gamma common (IL-2Rγc) chain is the shared signal transduction component of the IL-2R family, including IL-2R, IL-4R, IL-7R, IL-9R, IL-15R, and IL-21R, which each regulate aspects of immune development and function [1,2,3]. Inactivating mutations in human IL-2Rγc cause X-linked severe combined immunodeficiency (SCID), characterized by decreased numbers of T and NK cells and dysfunctional B cells, resulting in a significantly diminished immune response [4]. Mice lacking a functional IL-2Rγc display a broader form of X-linked SCID, with reduced T, NK, and B cells [5].

The zebrafish represents an important vertebrate model, with particular relevance to immunity [6,7]. Zebrafish undergo similar waves of hematopoiesis to mammals [8] and show broad conservation of cytokine receptor signaling components [9,10], particularly those known to facilitate the development of blood and immune cells [11,12,13,14,15]. We have previously identified two paralogs of the IL-2Rγc gene in zebrafish [9], and demonstrated that one of these, Il-2rγc.a, was involved in embryonic T-lymphopoiesis [16], and so potentially amenable to the generation of a zebrafish SCID model. Moreover, since zebrafish lack specific sex chromosomes [17], it was anticipated that this model would be sex-independent.

This research aimed to generate zebrafish *il2rga* mutants and characterize their lymphoid cells throughout the life-course, as well as investigate their microbiome and tumor immunity to confirm them as robust model of SCID.

## 2. Results

### 2.1. Generation of Il-2rγc.a Mutants

**Figure 1:**
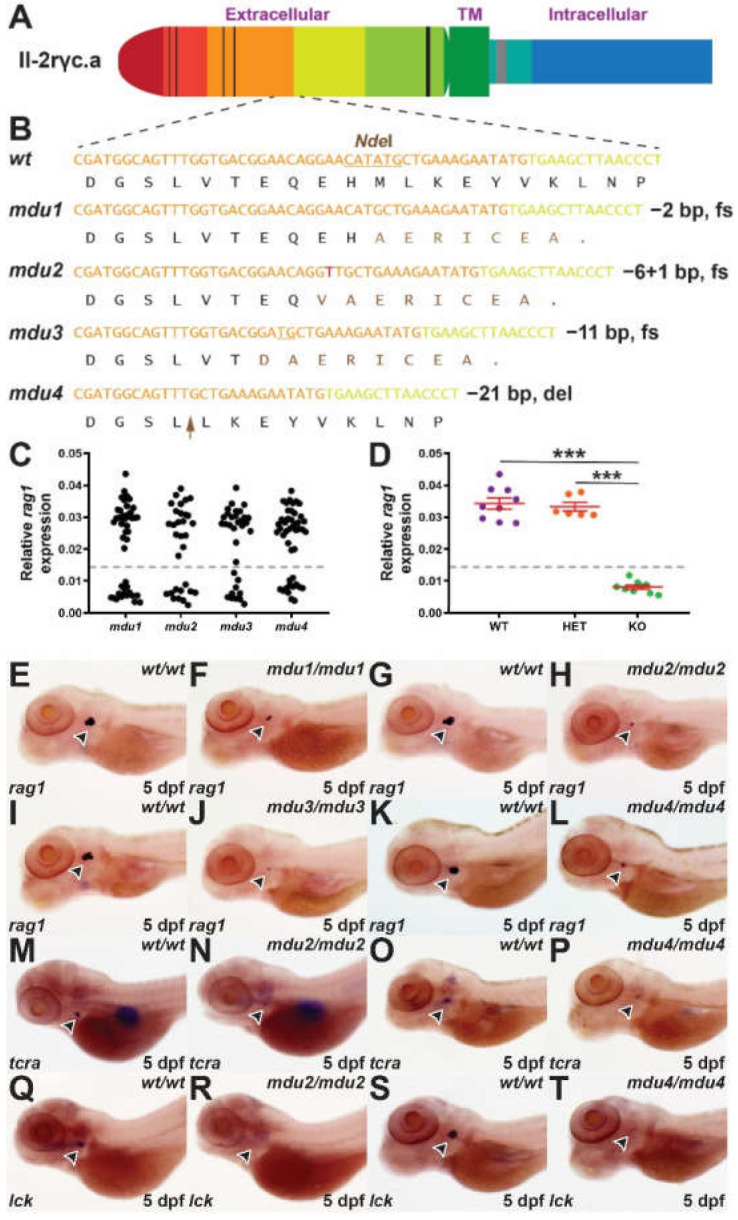
Identification of zebrafish *il-2rγc.a* mutant alleles and their impact on embryonic lymphopoiesis. (**A**) Schematic of zebrafish Il-2rγc.a with the extracellular domain, containing cysteine bonds (thin black lines) and a WSXWS motif (thick black line), shown to the left, followed by the transmembrane (TM) domain and the intracellular domain containing Box 1 (thick grey line), with alternate coloring depicting derivation from different exons. (**B**) Mutant *il2rga* alleles. Sequence of wild-type (*wt*) and indicated mutant (*mdu*) alleles, *il2rga^mdu1^*, *il2rga^mdu2^*, *il2rga^mdu3^*, and *il2rga^mdu4^*, along with their impacts at the nucleotide and protein level (fs, frameshift; del, deletion). Nucleotide sequences are colored according to their derivation from exon 3 (orange), exon 4 (yellow), or *de novo* (red), with the *Nde*I cut site used for genotyping underlined. The corresponding protein translations are shown below, with wild-type sequence in black and that derived from an alternative reading frame in brown, with the position of the seven residue deletion in *mdu4* indicated with the brown arrow. (**C**–**T**) WISH analysis. Embryos obtained from in-crossing of heterozygotes carrying the indicated *il2rga* alleles were fixed at 5 dpf and subjected to WISH with the indicated markers. Expression of *rag1* in individual embryos was quantified relative to eye size for each allele (**C**) (*n* = 31–46), and a selection of embryos genotyped by *Nde*I digestion to allow determination of their genotype (WT, wild-type; HET, heterozygote; KO homozygote mutant) (**D**), showing results for individual embryos, with mean and SEM in red and statistical significance indicated (***: *p* < 0.001, *n* = 6–9). Expression of *rag1* (indicated with arrowheads) in representative wild-type and homozygous mutant embryos derived from *il2rga^mdu1^* (*mdu1*) (**E**,**F**), *il2rga^mdu2^* (*mdu2*) (**G**,**H**), *il2rga^mdu3^* (*mdu3*) (**I**,**J**) and *il2rga^mdu4^* (*mdu4*) (**K**,**L**), *tcra* from *il2rga^mdu2^* (**M**,**N**) and *il2rga^mdu4^* (**O**,**P**) and *lck* from *il2rga^mdu2^* (**Q**,**R**) and *il2rga^mdu4^* (**S**,**T**) are shown.

The extracellular region of zebrafish Il-2rgc.a (Figure 1A) was targeted with TALENs designed to exon 3 of the *il2rga* gene as described [16]. Embryos injected with the TALENS were raised and then crossed with wild-type adults with multiple independent mutant alleles identified amongst their progeny using high-resolution melt analysis and verified by sequencing (Figure S1). Founder fish harboring these mutations were again outcrossed and then in-crossed to yield homozygous mutants, which were sequenced to fully characterize the respective mutations (Figure S1). This identified three mutant alleles, *il2rga^mdu1^* (2 bp deletion), *il2rga^mdu2^* (6 bp deletion + 1 bp insertion), and *il2rga^mdu3^* (11 bp deletion) that resulted in frameshifts leading to premature stops similar to a known human SCID mutation [18], and *il2rga^mdu4^* (21 bp deletion) that led to a 7 amino acid deletion in the extracellular domain of the Il-2rγc.a protein, which overlapped with residues affected in other human SCID mutations [18] (Figure 1B).

### 2.2. Impact on Embryonic Lymphopoiesis

Lymphocyte progenitors populate the zebrafish thymus from approximately 2.5 dpf to initiate T lymphopoiesis, which is well established by 5 dpf [19]. Therefore, heterozygote carriers of each *il2rga* allele were in-crossed and subjected to WISH with the T cell marker *rag1* [20] at 5 dpf. This identified a reduced area of expression in around 25% of the embryos in each case (Figure 1C), consistent with the expected Mendelian ratio for homozygous mutants. To confirm this, individual embryos were genotyped, which revealed no difference in *rag1* expression between heterozygous and wild-type embryos, but a significant decrease in expression in homozygous mutant embryos compared to the other genotypes (Figure 1D and data not shown), with a 100% correlation between low *rag1* expression and homozygosity for all mutant alleles (Figure 1E–L and data not shown). This analysis was extended to markers of mature T cells, *tcra* [21] and *lck* [22], for il2rga^mdu2^, as a representative of the frame shift mutants, and the deletion mutant il2rga^mdu4^. Substantially decreased expression of both markers was observed in homozygous mutants compared to wild-type siblings in each case (Figure 1M–T).

### 2.3. Impact on Later Lymphopoiesis

**Figure 2:**
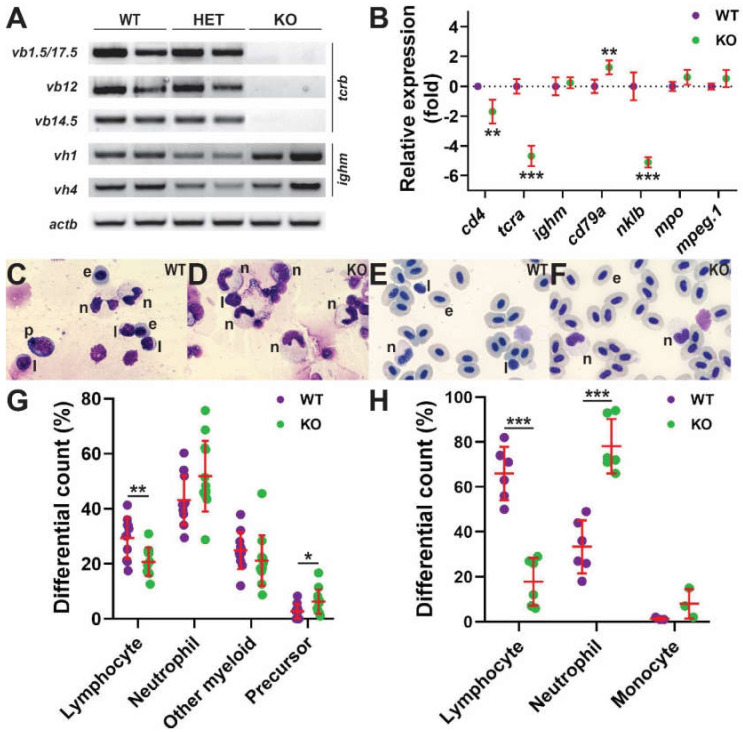
Effect of Il-2rγc.a ablation on later hematopoiesis. (**A**,**B**) Gene expression analysis. Larvae (28 dpf) obtained from in-crossing *il2rga^wt/mdu2^* heterozygotes were harvested for gDNA and total RNA. The gDNA was used for the identification of wild-type (WT), heterozygote (HET), and homozygote knock-out (KO) siblings, and the RNA was subjected to RT-PCR with primers specific for T cell receptor β chain V(D)J-Cβ rearrangements (*tcrb*: *vb1.5*, *vb12*, *vb14.5*), B cell immunoglobulin M heavy chain gene rearrangements (*ighm*: *vh1*, *vh4*), and β-actin (*actb*) as a control (**A**) RT-negative controls yielded no products (data not shown) (*n* = 2). (**B**) Adult kidney derived total RNA was subjected to qRT^2^-PCR with gene markers of T cells (*cd4*, *tcra*), B cells (*ighm*, *cd79a*), NK cells (*nklb*), neutrophils (*mpo*), and monocyte/macrophages (*mpeg1.1*) (**B**) Data was normalized relative to *actb* and represented as relative fold change compared to wild-type fish, with mean and SD shown in red and statistical significance indicated (**: *p* < 0.01, ***: *p* < 0.001, *n* = 6). Histological analysis (**C**–**H**) Representative images of Giemsa-stained kidney (**C**,**D**) and blood (**E**,**F**) cells were obtained from WT (**C**,**E**) and KO (**D**,**F**) fish. Abbreviations: e, erythrocyte; l, lymphocyte; n, neutrophil; p, precursor. Differential counts of kidney (**G**) and blood (**H**) cells, showing results for individual embryos, with mean and SD in red and statistical significance indicated (*: *p* < 0.05, **: *p* < 0.01, ***: *p* < 0.001, kidney: *n* = 11–12, blood: *n* = 6).

Zebrafish B lymphocytes develop from around three weeks post fertilization [23], with NK-related cells also readily detectable at this time [24]. Therefore, larvae from heterozygote in-crosses were individually collected at 28 dpf and both gDNA and RNA extracted. This allowed simultaneous genotyping and analysis of T cell receptor β (*tcrb*) and immunoglobulin M heavy chain (*ighm*) gene rearrangement, as markers of T and B cell maturation, respectively [25,26]. Wild-type and heterozygous mutant larvae derived from il2rga^mdu2^ showed equivalent *tcrb* rearrangement, but this was drastically reduced in homozygous mutants (Figure 2A), which was confirmed for other alleles (data not shown). In contrast, equivalent rearrangement of *ighm* was observed in wild-type, heterozygote, and homozygote siblings derived from *il2rga^mdu2^* and other alleles (Figure 2A and data not shown).

To investigate in further detail, the adult kidney, which serves a similar function to mammalian bone marrow with respect to hematopoiesis [27], was analyzed for the expression of genes specific for T cells (*cd4*, *tcra*), B cells (*ighm*, *cd79a*), NK cells (*nklb*), granulocytes (*mpo*), and monocyte/macrophages (*mpeg1.1*) [24]. Significantly decreased expression of all T cell markers was observed in homozygous mutants compared to wild-type siblings for both *il2rga^mdu2^* and other alleles (Figure 2B and data not shown). In contrast, B cell marker expression was equivalent or indeed slightly increased in homozygous mutants compared to wild-type siblings, whereas the NK cell marker was also decreased in homozygous mutants (Figure 2B and data not shown). There was similar expression of both leucocytic markers between wild-type and homozygote mutant siblings (Figure 2B and data not shown). Histological analysis of fish harboring the *il2rga^mdu2^* allele confirmed a significant decrease in lymphocytes overall in the kidney (Figure 2C,D,G) and particularly the blood (Figure 2E,F,H) of homozygous mutants compared to wild-type controls, with mutants showing significant elevation of precursors in the kidney (Figure 2C,D,G) and neutrophils in the blood (Figure 2E,F,H).

### 2.4. Impact on Intestinal Microbiota

**Figure 3:**
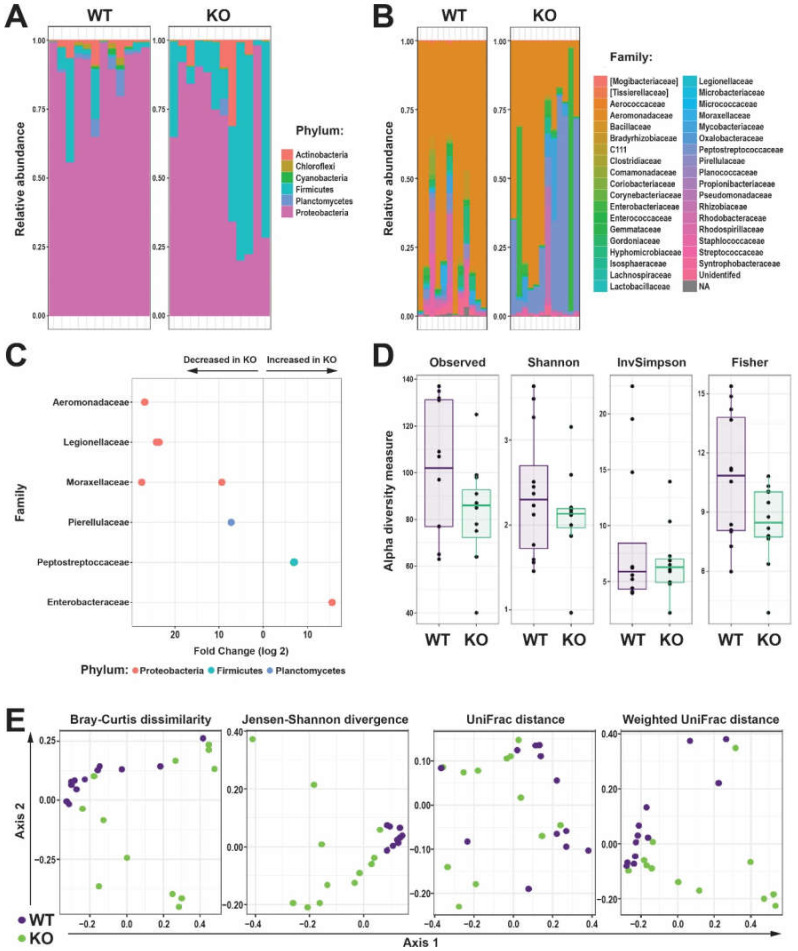
Effect of Il-2rγc.a ablation on intestinal microbiota. (**A**,**B**) Relative abundance of bacterial Amplicon sequence variants (ASVs) identified to the taxonomic level of phylum (**A**) and family (**B**) in the intestinal microbiome of individual WT (purple) and KO (green) zebrafish. (**C**) Differential abundances of bacterial family statistically different (*p* < 0.05) between WT and KO zebrafish, with the direction of change indicated. (**D**) Alpha diversity of WT and KO zebrafish intestinal microbiome displaying Observed, Shannon, InvSimpson, and Fisher’s indices as box and whisker plots. (**E**) Beta diversity of WT (purple) and KO (green) zebrafish intestinal microbiome displaying Bray-Curtis dissimilarity, Jensen-Shannon divergence, Unifrac, and Weighted Unifrac of log-transformed relative abundances ordination plots, with each dot representing an individual fish (*n* = 12 each genotype).

Strong reciprocal interaction exists between the microbiota and the immune system [28], with the microbiota known to be disrupted in immune compromised individuals [29], including those with SCID [30]. Therefore, we investigated the intestinal microbiota in wild-type (*WT*, *il2rga^wt/wt^*) and knock-out (*KO*, *il2rga^mdu2/mdu2^*) zebrafish. The majority of bacterial Amplicon sequence variants (ASVs) in the intestinal microbiome from fish of both genotypes were represented by the phyla Proteobacteria, although this was higher in WT (87.4%) compared to KO (64.0%) zebrafish. In contrast the relative abundance of the phylum, Firmicutes was greater in KO (30.2%) compared to WT (6.3%) zebrafish (Figure 3A). Differences were similarly noted at the family level, with the relative abundance of Aeromonadaceae substantially greater in WT (75.7%) compared to KO (45.4%) zebrafish (Figure 3B). Analysis of differential abundance of individual ASVs identified several that were significantly different between WT and KO zebrafish. Specific members of the Aeromonadaceae, Legionellacea, Moraxellaceae, and Pirellulaceae families were present at significantly lower abundance in KO compared to WT, while members of the Peptosteptococcacea and Enterobacteriaceae families were found to be present at a significantly higher abundance in KO compared to WT zebrafish (Figure 3C).

Analysis of alpha diversity indices, which describe ecological diversity within microbial community samples, revealed a significant reduction in KO compared to WT zebrafish, which reached significance using the Fisher index (Figure 3D). Analysis of beta diversity revealed significant differences between the microbial communities of WT and KO zebrafish, with genotype accounting for 31.9% (Jensen-Shannon divergence; *p* = 0.001), 15.8% (Bray-Curtis dissimilarity; *p* = 0.002), 18.7% (Weighted Unifrac distance; *p* = 0.012), and 14.6% (Unifrac distance; *p* = 0.014) of variance (Figure 3E).

### 2.5. Impact on Anti-Tumor Immunity

**Figure 4:**
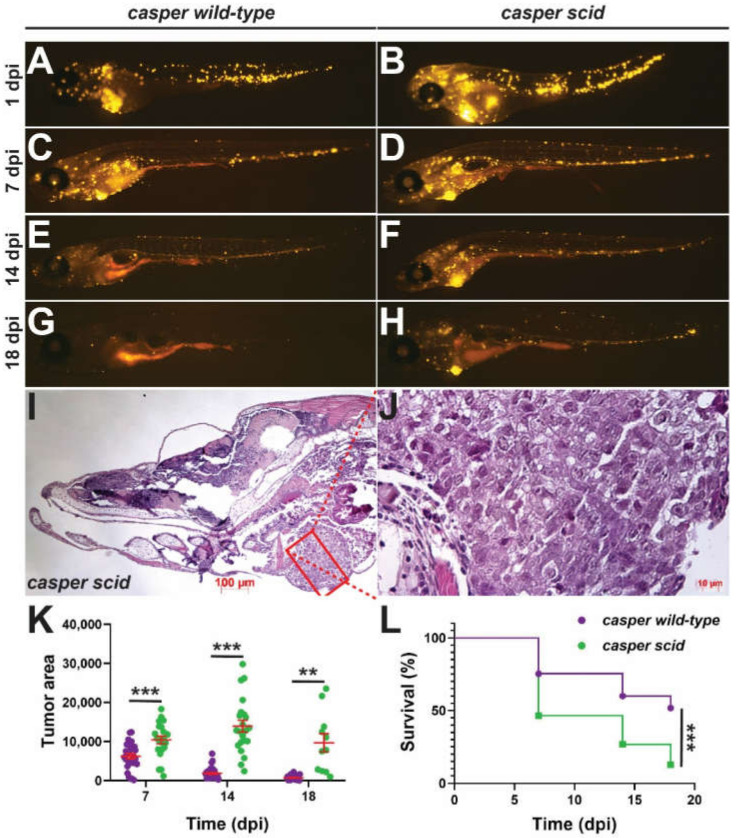
Effect of Il-2rγc.a ablation on tumor immunity. (**A**–**H**) Representative *casper* fish harboring *il2rga^wt/wt^* (*casper wild-type*) (**A**,**C**,**E**,**G**) or *il2rga^mdu2/mdu2^* (*casper scid*) (**B**,**D**,**F**,**H**) injected with fluorescently-labeled human HCT116 colorectal cancer cells imaged at the indicated days post injection (dpi). (**I**–**I’**. Histology of a representative tumor from a *casper scid* fish at 18 dpi at low magnification (5×, **I**) and of the boxed area at high magnification (40×, **J**). (**K**) Quantitation of tumor area showing results for individual embryos, with mean and SEM in red. (**L**) Kaplan–Meier survival curve of transplanted fish. For panels (**K**–**L**), statistical significance is indicated (**: *p* < 0.01, ***: *p* < 0.001, *n* = 11–24).

The large changes in lymphocyte populations and altered microbiome were consistent with significant compromise of the immune system. This was directly tested by examining the growth of xenotransplanted human tumor cells. To facilitate these studies, the *ilrga^mdu2^* allele was crossed onto the *casper* mutant, which lacks pigmented melanophores and iridophores greatly enhancing transparency [31] (Figure S2). Groups of *il2rga^wt/wt^* and *il2rga^mdu2/mdu2^* embryos on the *casper* background were injected with fluorescently-labeled human HCT116 colorectal cancer cells and monitored at regular intervals. This revealed the extent of the fluorescence was greater and more sustained in *il2rga^mdu2/mdu2^* compared to il2rga^wt/wt^ juveniles (Figure 4A–H), with tumors clearly evident in 18 dpi *il2rga^mdu2/mdu2^* individuals (Figure 4I,J). Quantification of fluorescence confirmed that the tumor burden was significantly higher across all timepoints (Figure 4K), with survival of transplanted *il2rga^mdu2/mdu2^* fish significantly reduced (Figure 4L).

## 3. Discussion

Multiple mutant alleles of zebrafish *il-2rγc.a* were successfully generated using TALEN-based genome editing, three of which resulted in frameshifts leading to premature stops in a similar position to the known human A156fs SCID mutation [18], while the fourth led to a 7 amino acid deletion that overlapped with those impacted in other human SCID mutations [18]. Both classes of mutations impacted T and NK lymphopoiesis, demonstrating a conservation of receptor structure/function. Homozygous knockout embryos showed severely decreased expression of multiple T cell markers at 5 dpf compared to heterozygous mutants and wild-type embryos. Analysis at 28 dpf revealed abrogation of TCRβ, but not IgM rearrangement in knockouts, while adult knockouts displayed lymphopenia in the blood and kidney, with reduced expression of T and NK cell markers, but strong expression of B cell markers. As expected, no differences were observed between male and female fish (data not shown). Collectively, this indicates the Il-2rγc.a mutants present with an autosomal T-B + NK-SCID.

The impact of IL-2Rγc ablation has now been studied in multiple species, which has revealed phenotypic differences. Mice and rabbits lacking functional IL-2Rγc have a more severe phenotype than humans, with B cells also severely reduced [5,32]. However, pigs in which IL-2Rγc is ablated are more similar to humans with T and NK cells affected [33], similar to what we have observed in the zebrafish *il2rga* mutants.

Alternate zebrafish SCID models have been described. Homozygote *rag1* mutants lacked mature T and B cells, but showed comparable expression of NK cell markers to wild-types indicative of a T-B-NK + SCID phenotype, consistent with their human equivalent [26]. Homozygote *prkdc* mutants showed a similarly severe phenotype [34]. However, homozygote *rag2* mutants exhibited a less severe immunodeficiency, with a strong decrease in mature T cells, but with B cells only moderately affected [35]. In contrast, homozygote *cmyb* mutants showed severely reduced numbers of T, B, NK, and myeloid cells, and unlike the other mutants failed to survive to adulthood [36].

Human SCID is associated with opportunistic infection and chronic diarrhea [37], with SCID patients showing reduced microbial diversity [30]. This has also been observed in animal models, with SCID (and NOD/SCID) mice showing reduced alpha diversity, with a concomitant increase in SCFA-producing bacteria [38]. The main butyrate producing-bacteria in the human gut belong to the phylum Firmicutes, which was also substantially increased in the zebrafish *il2rga* mutants. This indicates that our zebrafish model faithfully reproduces this aspect of SCID biology. Further analysis of the microbiome and its interaction with the immune system of zebrafish *il2rga* mutants could provide new insights.

Zebrafish have proven to be a useful model for transplantation studies, due to their relative ease of manipulation and visual clarity compared to traditional rodent models. Many of these studies have used wild-type embryos prior to the onset of adaptive immunity [36]. However, immunocompromised lines are becoming more widely used. For example, *rag2* knockout zebrafish have been used to visualize tumor neovascularization [24] while *prkdc* knockouts have supported long-term engraftment of xenotransplanted gastrointestinal cancer cells [34]. The zebrafish *il2rga* mutants on the *casper* background showed reduced tumor immunity, allowing successful tumor engraftment in 86.4% of embryos at 14 dpi, compared to 8.6% in wild-type controls.

This paper describes the generation and characterization of zebrafish *il2rga* mutants. They exhibited a robust SCID phenotype, including a reduction in T and NK cells but not B cells, as well as an altered microbiome and defective tumor immunity. It is anticipated that this model will prove applicable to patient-derived xenografts, which has been demonstrated to be an effective platform for the evaluation of cancer therapy in a patient-specific manner [39], with additional application to the further exploration of SCID-microbiome interactions.

## 4. Materials and Methods

### 4.1. Zebrafish Husbandry and Manipulation

Wild-type and *casper* [31] zebrafish were maintained using standard husbandry practices [40]. This included feeding thrice daily with a mixture of live feed (artemia and rotifers) and a dry granulated foodstuff (Otohime Hirame Japan). Embryos were injected with 100 pg/nL mRNA encoding transcription activator-like effector nucleases (TALENs) [41] targeting an *Nde*I site exon 3 of *il2rga*, as described [16] and raised to adulthood. Progeny were screened for the presence of mutations by PCR of genomic DNA derived from fin-clips with primers flanking the target site (5′-CGAAGACTGTCCTGAATATGAGAC; 5′-TCTGGTCAGTCCTGTAACGAAC) followed by high resolution melting (HRM) analysis using Precision Melt Suremix and Analysis Software (BioRad, Hercules, CA, USA) [42] and confirmed by restriction fragment length polymorphism analysis with *Nde*I as well as Sanger sequencing (Australian Genome Research Facility, Melbourne, Australia). Confirmed mutants were out-crossed with wild-type fish for two generations with heterozygote progeny ultimately in-crossed to generate homozygous mutants or crossed onto the *casper* background.

### 4.2. Reverse-Transcription Polymerase Chain Reaction

Total RNA was extracted from zebrafish embryos, larvae, and pooled adult zebrafish tissues with RNeasy Mini Kit (Qiagen Pty Ltd Australia, Clayton, Australia) according to the manufacturer’s protocol for animal tissues. This was subjected to semi-quantitative reverse-transcription polymerase chain reaction (RT-PCR) with primers for T-cell receptor beta (TCRβ) chains vb1.5/17.5 (5′-AATGGACAGCTTGATAGAACTGAAC, 5′-TGCTTATTCAACCGAACAGAAACATTC), vb12 (5′-CAGACACCGTGCTTCAGTCGAG, 5′-ACGTTTCATGGCAGTGTTACCTG) and vb14.5 (5′-GAATCCAATGTGACGTTAACATGC, 5′-CATGATCATAAGGACCACTACAG) and immunoglobulin M heavy chains vh1 (5′-GATGGACGTGTTACAATTTGG, 5′-CCTCCTCAGACTCTGTGGTGA) and vh4 (5′-CAAGATGAAGAATGCTCTCTG, 5′-TGTCAAAGTATGGAGTCGA) or quantitative real-time RT-PCR (qRT^2^-PCR) with *actb* (5′-TGGCATCACACCTTCTAC, 5′-AGACCATCACCAGAGTCC), *cd4* (5′-TCTTGCTTGTTGCATTCGCC, 5′-TCCCTTTGGCTGTTTGTTATTGT), *tcra* (5′-ACTGAAGTGAAGCCGAAT, 5′-CGTTAGCTCATCCACGCT), *ighm* (5′-CCGAATACAGTGCCACAAGC, 5′-TCTCCCTGCTATCTTTCCGC), *cd79a* (5′-GCGAGGGTGTGAAAAACAGT, 5′-CCCTTTCTGTCTTCCTGTCCA), *nklb* (5′-TGCTGCGCGGTATCGTC, 5′-GCACACATGGAGATGAGAACAG), *mpo* (5′-CTGCGGGACCTTACTAATGATG, 5′- CCTGGATATGGTCCAAGGTGTC) and *mpeg1.1* (5′-CACCTGCTGATGCTCTGCTG, 5′-CCAGACCTCCCAACACCAAC). Data were normalized to *actb* and fold change was calculated using the ΔΔCt method.

### 4.3. Whole-Mount in Situ Hybridization (WISH)

Embryos were dechorionated and fixed in 4% (*w*/*v*) paraformaldehyde (PFA) at 4oC prior to WISH with DIG-labeled anti-sense probes, as described [43]. Quantitation was achieved by measuring the area of staining relative to eye diameter as determined using CellSens Dimension 1.6 software (Olympus) on approximately 30 embryos in a blinded fashion.

### 4.4. Ex Vivo Analysis

Cytospin preparations were prepared from adult blood and kidney and stained with Giemsa (Sigma-Aldrich, North Ryde, Australia), and differential counts performed. Alternatively, adult zebrafish kidney cells were prepared in ice-cold phosphate-buffered saline supplemented with 2 mM EDTA and 2% (*v*/*v*) fetal calf serum and passaged through a 40 μm sieve and analyzed using a FACSCantoII with cell populations identified in a side-scatter (SSC)/forward-scatter (FSC) plot.

### 4.5. Tumor Xenotransplantation

Human colorectal cancer cells HCT116 were fluorescently labelled with Vybrant Dil cell labelling solution (cat#V22885, Invitrogen, Thermofischer Scientific, Scoresby, Australia) according to the manufacturer’s instructions. Labelled cells were washed twice in PBS and resuspended in DMEM media at a concentration of 3–4 × 10^5^ cells/μL, and approximately 1–2 × 10^3^ cells microinjected into the perivitelline space of anesthetized 48 hpf zebrafish embryos using borosilicate glass micro capillary needles. Embryos with adequate transplanted cells and normal morphology were transferred to fresh E3 medium to recover and held at 33.5 °C, with fluorescent microscopy used to visualize tumor cells, with engraftment and survival monitored. At 18 dpi, the fish were euthanized and fixed overnight in 4%PFA/PBS before embedding in paraffin for sectioning (3 μm), with the sections stained with Hematoxylin and Eosin.

### 4.6. Imaging

Imaging of was performed using Olympus MVX10 fluorescence microscope and DP72 camera using Cellsens Dimension 1.6 software, except for histological sections that were imaged using a Zeiss Axio Imager 2 microscope. ImageJ was used for quantification analysis, with analyze particles plugin used for determining the total number of tumor cells and the total tumor area.

### 4.7. Statistical Analysis

Statistical analysis were performed using Graph Pad Prism software, version 8. Student’s *t*-tests were performed for statistical analysis of the data, with Welch’s correction done where necessary. Survival was plotted as a Kaplan–Meier curve, with statistical significance determined using a log-rank (Mantel–Cox) test.

### 4.8. Microbiome Analysis

Dissected zebrafish intestines were cut finely before isolation of genomic DNA using a DNeasy blood and tissue kit (Qiagen Pty Ltd Australia, Clayton, Australia). 16S library preparation and sequencing were performed at Deakin Genomics Centre (Victoria, Australia). All primary PCRs were performed in triplicate with 5 μL NEB Q5 hi-fidelity hot start 2× mastermix, 1.25 μL of each primer (1 μM), and 2.5 μL DNA (2 ng/μL) or water. PCR conditions were 98 °C for 3 min, then 25 cycles of 98 °C for 30 s, 55 °C for 30s, and 73 °C for 45 s. A final extension at 72 °C for 5 min performed. A total of 2 μL of each sample was run on a 1.5% TAE agarose gel to confirm amplification. The triplicates were pooled and cleaned with 1 volume of Ampure beads and resuspended in 20 μL ultrapure water. Indexing was performed with 5 μL NEB Q5 hi-fidelity hot start 2× mastermix, 1.25 μL each index primer (5 μM), and 2.5 uL of DNA or water. PCR conditions were 98 °C for 3 min, then 6 cycles of 98 °C for 30 s, 57 °C for 30 s, and 73 °C for 45 s, with a final extension at 72 °C for 5 min. The indexed PCR products were visualized on a 1.5% agarose TAE gel. A 5 μL aliquot of each library was pooled and 25 μL cleaned with 1 volume of Ampure beads, with the concentration checked with a Quibit HS assay and size confirmed with Tapestation HS D1000 tape. The indexed library pool was diluted to 4 nM with ultra-pure water. The 4 nM pool concentration was checked by qubit HS DNA assay to verify accurate dilution. A total of 5 μL of the 4 nM pool was denatured for 5 min with 5 μL fresh, 0.2N NaOH. A total of 990 μL of HT1 buffer was added to create 1 mL of 20 pM library. A 10% volume of 20 pM phiX was added to add diversity. The pool was further diluted to 6 pM with HT1 buffer. Sequencing was performed on 600 μL of this with a MiSeq sequencer using V3 (3 × 300 bp) chemistry.

Statistical analysis on the bacterial microbiome was performed in R. Relative abundance of bacteria was visualized in different sample types, classified to the phylum and family levels. To identify differences in the bacterial microbiome between WT and KO groups, permutated multivariate analysis of variance (PERMANOVA) was conducted. The R package DESeQ2 [44] was used to identify taxonomic units that were differentially abundant within groups. Results for statistical tests were considered to be significant, where *p* values < 0.05. For DESeQ2 results, the threshold for statistical significance was a false detection ratio (FDR) < 0.05. Alpha diversity using Observed, Shannon, InvSimpson, and Fisher’s diversity indices were computed using the richness function in the package phyloseq [45] and visualized using boxplots with the phyloseq and ggplot2 packages. Differences were tested with the Welch two-sample *t*-test for the Shannon, Observed and Fisher indices, and Wilcoxon rank sum exact test for the InvSimpson index. The final Amplicon sequence variant (ASV) dataset was filtered using the Callahan workflow [46]. Beta-diversity was evaluated with Bray-Curtis, Jensen-Shannon, and UniFrac and were visualized using Principal Coordinate analysis (PCoA) plots with phyloseq.

## Data Availability

All data generated or analyzed during this study are included in this published article (and its Supplementary Materials).

## Supplementary Materials

The following supporting information can be downloaded at: https://www.mdpi.com/article/10.3390/ijms23042385/s1.
