# Supplementary material for: Generation and Characterization of a Zebrafish IL-2Rγc SCID Model
Source: Int J Mol Sci. 2022 Feb 21;23(4):2385. doi: 10.3390/ijms23042385 (PMC8875600; doi:10.3390/ijms23042385)
Supplement: Supplementary file 1 [file ijms-23-02385-s001.zip › zf il2r ko ijms Supp Fig 1 SCID.pdf]

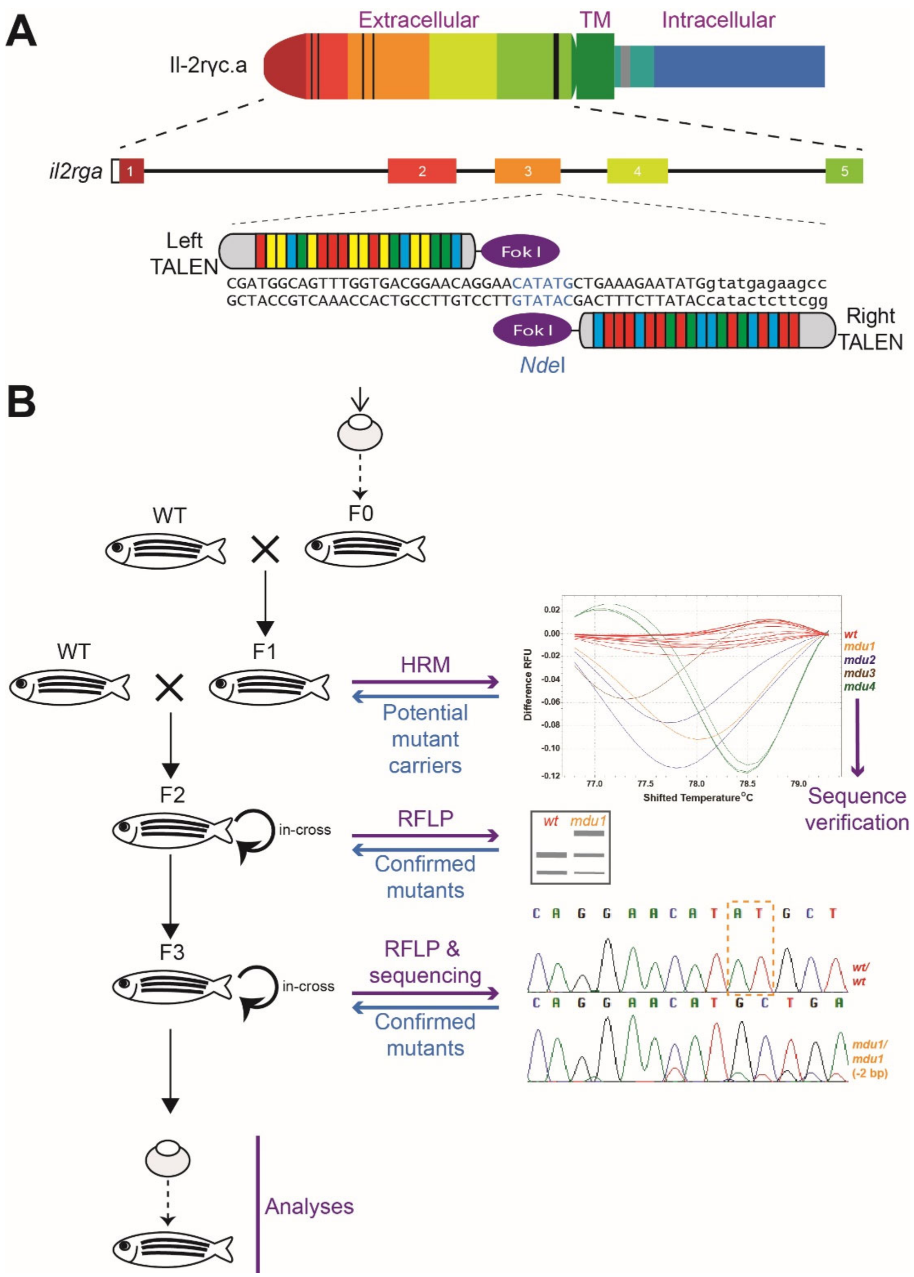

Figure S1: Targeting il2rga with TALENs.

A. Schematic representation of Il-2rγc.a as detailed in Figure 1A, with exons corresponding to the extracellular domain shown below numbered and boxed and the sequence targeted by each TALEN displayed further below. Exonic sequences are in uppercase, intronic sequence in lower case and the *Nde*I site shown in blue, with the TALEN binding domains color-coded based on nucleotide binding (T: red, G: yellow, C: blue, A: green) and the *Fok*I endonuclease shown in purple.

B. TALEN mRNA was injected into 1 cell stage fertilized zebrafish embryos which were raised to adulthood with these founder (F0) fish then crossed with wild-type (WT) fish. F1 progeny were screened using high resolution melt (HRM) analysis to identify and differentiate between various *il2rgca* alleles including wild-type (*wt*) and various mutants (*mdu*) as indicated, with potential mutants sequence verified. These were then out-crossed to WT fish with F2 carriers identified using RFLP analysis with *Nde*I, which were subsequently in-crossed to yield F3 mutants that were identified by RFLP analysis and confirmed with sequencing, as shown for homozygous *wt* and *mdu1*. Analyses were performed on progeny of confirmed mutants, with progeny of wild-type fish also used as a source of controls.
