# Supplementary material for: Generation and Characterization of a Zebrafish IL-2Rγc SCID Model
Source: Int J Mol Sci. 2022 Feb 21;23(4):2385. doi: 10.3390/ijms23042385 (PMC8875600; doi:10.3390/ijms23042385)
Supplement: Supplementary file 1 [file ijms-23-02385-s001.zip › zf il2r ko ijms Supp Fig 2 Casper.pdf]

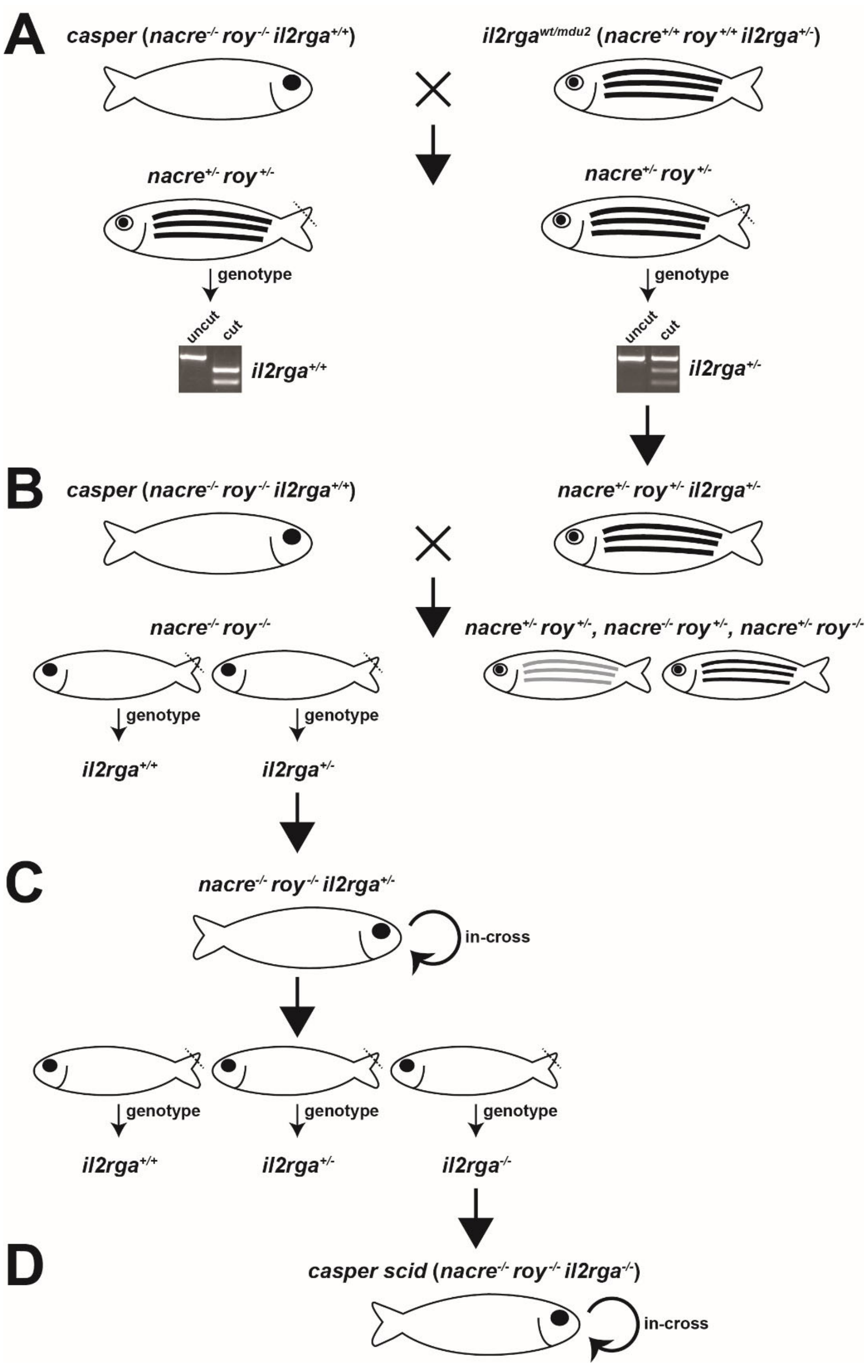

Figure S2: Generation of combined casper scid mutant.

Schematic representation of the generation of an *il2rga* (*scid*) mutant on the *casper* background. A. Transparent *casper* zebrafish, containing homozygote mutations (-/-) in *nacre* and *roy* but wild-type (+/+) for *il2rga*, were crossed with heterozygote (+/-) *il2rga*$^{wt/mdu2}$ mutants, which were wild-type with respect to *nacre* and *roy*. Progeny were all heterozygous for *nacre* and *roy*, with those being *il2rga*$^{+/-}$ identified by genotyping of fin clip-derived genomic DNA using RFLP analysis with *Nde*I, with representative images of undigested (uncut) and digested (cut) PCR products from *il2rga*$^{+/+}$ and *il2rga*$^{+/-}$ fish. B. Transparent *casper* fish were then crossed with fish heterozygous for *nacre*, *roy* and *il2rga*. Progeny were visually inspected for total lack of pigmentation to identify homozygote *nacre* and *roy* fish, which were genotyped to identify *il2rga*$^{+/-}$ fish. C. These were then in-crossed, and progeny genotyped to identify *il2rga*$^{-/-}$ (*scid*) mutants on the *casper* background. D. This *casper scid* line was subsequently maintained by in-crossing.
